# Medical Observations in the Clifton Zoological Gardens, with Notes on the Birth of Two Russian Bear Cubs in Captivity

**Published:** 1913-12

**Authors:** A. J. Harrison

**Affiliations:** Consulting Physician to the Bristol General Hospital.


### MEDICAL OBSERVATIONS IN THE CLIFTON ZOOLOGICAL GARDENS, WITH NOTES ON THE BIRTH OF TWO RUSSIAN BEAR CUBS IN CAPTIVITY.

BY

A. J. HARRISON, M.B. Lond.,

*Consulting Physician to the Bristol General Hospital.*

---

SOME years ago, I gave to the Bristol Medico-Chirurgical Society a presidential address, which I designated as "Comparative Pathology."[1] It related chiefly to some of the diseases of animals as met with by me in our Zoological Gardens in Clifton. In those days but little was known and recorded of this branch of Comparative Pathology, which has now rightly assumed a very important position.

Time has gone on, and I have been asked if I would give a few more details. I am quite sure that most of us take an interest in animals, be they horses, dogs, cats, pigeons, canaries, etc., and are accustomed to give pet names to our favourites, and thus to identify them more closely with our own surroundings. In a very similar way we give—in our zoological collections—more or less familiar names to *wild* animals, and thus take them somewhat nearer to our affections. "Sallie" to a celebrated chimpanzee, "Jumbo" to a world-renowned elephant, and "Alice" to his companion, were names well-known a few years ago in our Regent's Park Gardens in London. "Hannibal," "General," "Jupiter," "Prince," "Queenie," "Victoria," have been and are (most of them) familiar in our own Clifton Zoo, and quite recently there has been an exciting competition over the name of the new Indian elephant, when "Rajah" won the day. The celebrated actress who has

[1] *Bristol M-Chir. J.*, 1894, xii. 273.

quite recently been amongst us, Sarah Bernhardt, had as a rather close companion a favourite tigress!

Well, these animals are subject to many complaints that human flesh is heir to. They have decayed teeth, and inferentially tooth-ache. They have enlarged internal organs, "liver," "spleen," etc., and in-growing claws, corresponding to our in-growing toe and finger-nails; inflammation of the lungs, "pneumonia"; inflammation of the covering of the lungs, "pleurisy" and "empyema," or of the bag of the heart, "pericarditis," or of the stomach, "gastritis," or of the intestines, "enteritis," etc., etc.

Nor are they free from some forms of cancer, of swellings and tumours. Our domestic pets are subject to like ailments, or they may be the means of conveying infection, measles, scarlet fever, ring-worm, even diphtheria, and a form of infectious pneumonia by going from room to room, or from one house to another. A few years ago a painful sensation was created in Paris and its vicinity by an infectious and very fatal form of pneumonia having been traced in its origin to a malady amongst parrots.

In my previous paper I gave an account of "Jupiter," a favourite lion, having an in-growing claw removed. The account got into the papers, and *The Standard* gave a long editorial and raised the question whether the necessity for operations arose amongst wild animals in their natural state, and also of what became of the bones of dead animals, for they were so seldom and so scantily found by hunters and travellers. Let me throw, if possible, a little light upon this interesting question. We had a hyena in the Zoo for many years, and this animal would grind up and consume the hardest bones which could be given to it.

Let us reflect for a few moments what this crushing power means. What power is exerted by the jaws of a hyena in breaking up say the leg-bone of a horse or the jaw-bone of a bullock? Experiments which were made with very great care and precision demonstrated that a crocodile weighing 154 lb. exerted a force of 308 lb. in snapping its jaws. The power exerted by a hyena must have been very much more. We are in the habit of praising the strength of a man by describing him "as strong as a horse," or "an elephant," or "a lion!" If we compared him to a cockchafer, a bee, or a flea, we should exalt his power much more. Experiments have shown that a flea can jump two hundred times its height, a cockchafer can draw fourteen times its own weight, and a bee twenty times; or, in other words, a man 6 ft. high, if as strong as a flea, would be able to jump over a building three times the height of St. Paul's Cathedral!

It does not fall to the lot of everyone to extract a tooth from a tiger's mouth, even if the animal, as in this case, was only twelve months old. In the year 1887 we had a very fine tiger cub born in the gardens, but artificial rearing did not, possibly, suit its dentition, for it suffered much from changing its teeth. It began to refuse food, and then fell off in consequence. We determined to help the process of tooth removal, so one morning two keepers managed to get the animal into a strong sack. The sack was securely fastened round its neck, allowing the head to protrude. The "bagged" creature was passed between a keeper's legs, and the end of the bag held by another man. The tiger opened its mouth in a kind of self-defence, and this gave the opportunity for a third man to place a roll of wood between its jaws, and hold the roll from behind. In this way the animal was securely firm, and its mouth well opened, so that access to the teeth was thus made available. With a pair of strong tooth forceps the offending teeth were speedily and effectually removed, and presented to a medical friend who witnessed the proceedings. The gaping mouth looked rather formidable, but the operation was successful, and the patient much better for it.

It has often been remarked, "Why don't you give the animals an anæsthetic when an operation has to be performed?" The answer is that all these creatures are very intolerant of chloroform and other anæsthetics, and besides there are many difficulties in the way of administration. The following case has been already printed, but it may be as well to repeat it here, because of a somewhat similar occurrence, where no anæsthetic was given. Some years ago a favourite cockatoo had been placed in the bird-house by its mistress because it was becoming blind from cataract. Mr. Richardson Cross very kindly examined the eye, and an operation was determined upon. I gave the chloroform, and a piece of lint with only half a drachm of chloroform upon it was applied to the head of the bird, quite loosely. It could only have had an infinitesimal dose, when it fluttered its wings and died. Either its heart, which had responded again and again to the caresses of an over-indulgent mistress, would not be cajoled by the wiles of an anæsthetic, or its life-span coincided with the time of the administration, for birds often drop down dead from their perch. Let us corroborate this statement. We had at this time a white-necked buzzard—Buteo Albi-Collis—a very rare bird from Demerara, estimated to be worth £500. Some months after the death of the cockatoo this bird suddenly dropped down from its perch dead.

The felidæ are very prone to inflammation of the lungs. Almost every *post-mortem* upon them gives evidence of this, either as a recent condition, or as having occurred at a more remote period. Some years ago a male cheetah died of inflammation of both lungs—the right the more advanced—and then a few days afterwards a female cheetah died ; and not long afterwards a splendid male tiger succumbed to severe inflammation of the left lung, which had resulted in grey hepatisation.

In the autumn of 1894, I was in Hamburg, and whilst there visited Hagenbeck's famous collection of animals, and amongst them saw one of the rarest creatures the firm had ever come across, viz. a small-sized but full-grown white seal, an albino with distinctly pink eyes.

I also saw a very droll and rare sight, viz. a baby hippopotamus being fed with a bottle like a human infant. It had been brought from Egypt in a specially prepared water-tank. The rearing was quite successful, it grew up to maturity and may be still living.

Some years ago the Clifton Gardens lost quite suddenly and in a very extraordinary way a Brahmin bull, about five years old, and apparently in splendid condition. He was a great favourite, and had just been granted a new piece of park ground to add to his enjoyment. The season was a very dry one, and the keeper of the deer house was engaged in drawing water from the lake. About 3 p.m. he passed the bull, and saw him playing about in his demesne. In about two minutes he returned from the water and saw the creature lying dead, or nearly so, in his enclosure.

What had occurred ? Nothing but a *post-mortem* could have revealed the cause. Death was caused by fracture of the base of the skull. How did it happen ? We can only theorise, for there was no witness to the tragedy, the evidence was all circumstantial. The animal was very lively, and very fond of what is termed buck-jumping, *i.e.* throwing up his hind quarters whilst supporting himself on his fore legs. In doing this he must have slipped or over-balanced himself, and so have fallen with his head, or rather horns, crashing upon the ground, or more probably on the edge of the brick kerbing of his enclosure, for on careful examination we found the edge of the brick-work broken just where the roots of his horns in this position would impinge. In animals of this kind, and in bulls very especially, the occipital bone is very much developed, very thick and massive to give firm attachment to the neck muscles. In his case the bone was little thicker than an ordinary dinner plate, and the bone was fractured in several places.

During this last summer I paid a visit with some friends to Chillingham, the Earl of Tankerville's seat in Northumberland, and where the celebrated herd of wild white cattle are to be seen.

The day was calm and serene, and the hay harvest in full swing. In company with the head keeper, a very intelligent man named Elliot, we were conducted to the part of the park where the wild cattle happened to be. We had a most fortunate opportunity, for we got to within thirty yards of those nearest to us, and stood for quite half an hour. The unfortuntate thing was we had no camera with us, for it was a rare occasion for taking an excellent photo. Many of the cows lay chewing the cud, and some of the younger bulls were quietly sporting about, but were every now and then checked in their harmless festivities by the king bull. There is always a master or king bull, who holds his supremacy for a time, until he is dispossessed by a stronger animal, or is shot to give place to a more worthy successor. The herd at this time consisted of ninety-three members, and the ambition of the keeper was to get it up to a hundred.

These cattle are said to be the descendants of the original wild cattle of this country. They are kept quite distinct, and are never crossed with other cattle, or sold alive for interbreeding.

The peculiarities are these: they are fair-sized, white in colour except a black tuft to the tail, and have chestnut-tinted hairs about their ears. They have black muzzles, and the bulls have black tips to their horns, which, except this, are white. The cows have white horns, pointing forwards at first, then curving inwards and gradually more backwards as they advance in years. The bulls rarely come to perfection until they are four or five years old, and the cows rarely calve under four years. The bulls, if not destroyed and this mostly by shooting, live to a very great age for cattle. Thus a bull has been known to attain fifty-two years, and cows between thirty and forty years.

This longevity and slow arriving at pro-creation are very different to our more domestic cattle, and corresponds very much with our felidæ in the Zoo. The lionesses and tigresses rarely have cubs before they are four or five years old, and the males care little for pro-creation until attaining the same ages. Wild stags and deer, I understand from inquiries amongst experts and authorities on the subject, follow very much the same rule. Thus Mr. Archibald Hamilton says (*The Red Deer of Exmoor*): "It is a point much disputed whether a hind has her first calf at three or four years old . . . but when once she begins to breed she rarely misses a year until she attains a great age . . . and young male deer keep company with the hinds until they are about three years old, when they join the stags."

A friend who has had a large experience of stag-hunting, and more especially so in Scotland, thoroughly corroborates the above views, and also suggests that young stags would stand very little chance against the older ones until they had well-developed horns. Doubtless there is much to be said for this argument, but it does not convey the whole facts for lions and tigers in confinement, for there they are protected from any masculine competition. How different are our domestic cats—a kitten will often be a mother before she is a full-grown cat!

On Jan. 7th, 1913, or as near as can be ascertained, a Russian bear, "Judy," had two cubs born. Note the uncertainty of exact date. The reason of it is this. On Dec. 11th "Judy" went into retirement, or semi-hibernation, in a den which had been specially prepared for her, in the expectancy of her shortly afterwards becoming a mother. Cold country bears, as a rule, hibernate during the wintry months. They hide themselves away in caves, holes of trees, or make them a snug berth with boughs and leaves, or even a cavern in the snow itself. There they pass the cold days, feeding on their possession of fat. Now an expectant lady-bear cannot live altogether such an indolent life. The females always have their cubs in the winter, and therefore have to look after their progency as well as themselves, and *all* have to be sustained. The sustenance of all is the burden of fat which the expectant mother has been accumulating during the warmer weather. Well, "Judy" had her cubs, and then after a few days there were unmistakable sounds of infantine life, two cubs certainly, if not three were cradled. On March 27th she and her cubs were seen for the first time, but she had partaken of some food (which had been placed handy for her, and fairly continuously so) on the night of Feb. 24th, and she had also taken a little water apparently. It was a very important thing not to disturb her at all, for by interfering with their habits mothers have been known to destroy their cubs. After Feb. 24th she took food and water occasionally, but never voraciously; but even this small supply of nutriment had the good effect of preventing her from being very nearly reduced to a skeleton, covered with skin and hair, a condition which is stated to be the case when all around them is natural. Well, she showed herself then in fair condition, but the cubs were splendid, fat, well-formed, and decidedly jolly. No "teddy-bears" dressed up to delight the child-world have ever approached them. Their coats were absolutely clean and sleek, of a dark dove colour, whilst around their neck and shoulders was a white tippet, and along the back a dark brown stripe. "Judy" displayed the greatest care and anxiety in their behalf, and for weeks afterwards could not bear to have them out of her sight.

Even after she had brought them out for public gaze, for many days she still preferred their original den, although another one had been fitted up for a nursery; but when the opportunity did come to allow inspection, her maternal bed of peat moss was scrupulously dry and clean, but a short distance away was a place which had evidently served as a cloaca.

These facts are certain, viz. that for seventy-five days the mother had no food nor drink whatever, and she supported herself and her cubs on her own store of fat. The cubs have thrived well, and are most interesting and attractive, and "Judy" has long and well recovered from her protracted seclusion. None of our fasting heroes and heroines can hold a candle to "Judy"!

---

